# Progressive microstructural changes of the occipital cortex in Huntington’s disease

**DOI:** 10.1007/s11682-018-9849-5

**Published:** 2018-02-28

**Authors:** Omar F. F. Odish, Robert H. A. M. Reijntjes, Simon J. A. van den Bogaard, Raymund A. C. Roos, Alexander Leemans

**Affiliations:** 1 Department of Neurology (J3-R-162), Leiden University Medical Center, P.O. Box 9600, 2300 RC Leiden, The Netherlands; 2 Image Sciences Institute, University Medical Center Utrecht, Utrecht, The Netherlands

**Keywords:** Huntington’s disease, Premanifest, Diffusion tensor imaging, Longitudinal biomarker, Occipital cortex

## Abstract

In this study we longitudinally investigated the rate of microstructural alterations in the occipital cortex in different stages of Huntington’s disease (HD) by applying an automated atlas-based approach to diffusion MRI data. Twenty-two premanifest (preHD), 10 early manifest HD (early HD) and 24 healthy control subjects completed baseline and two year follow-up scans. The preHD group was stratified based on the predicted years to disease onset into a far (preHD-A) and near (preHD-B) to disease onset group. Clinical and behavioral measures were collected per assessment time point. An automated atlas-based DTI analysis approach was used to obtain the mean, axial and radial diffusivities of the occipital cortex. We found that the longitudinal rate of diffusivity change in the superior occipital gyrus (SOG), middle occipital gyrus (MOG), and inferior occipital gyrus (IOG) was significantly higher in early HD compared to both preHD and controls (all p’s ≤ 0.005), which can be interpreted as an increased rate of microstructural degeneration. Furthermore, the change rate in the diffusivity of the MOG could significantly discriminate between preHD-B compared to preHD-A and the other groups (all p’s ≤ 0.04). Finally, we found an inverse correlation between the Stroop Word Reading task and diffusivities in the SOG and MOG (all p’s ≤ 0.01). These findings suggest that measures obtained from the occipital cortex can serve as sensitive longitudinal biomarkers for disease progression in preHD-B and early HD. These could in turn be used to assess potential effects of proposed disease modifying therapies.

**Electronic supplementary material:**

The online version of this article (10.1007/s11682-018-9849-5) contains supplementary material, which is available to authorized users.

## Introduction

Huntington’s disease (HD) is a rare autosomal dominant neurodegenerative disorder caused by an expanded cytosine-adenine-guanine (CAG) repeat on chromosome 4. The hallmark feature in HD neuropathology is degeneration of the striatum. However, a growing amount of evidence from neuroimaging studies suggests that occipital regions are affected early on in the disease course (Tabrizi et al. 2009, 2012; Henley et al. 2009; Rosas et al. 2008; Hobbs et al. 2010; Muhlau et al. 2007; Coppen et al. 2016; Dogan et al. 2015; Ciarochi et al. 2016; Johnson et al. 2015; Harrington et al. 2016; Matsui et al. 2015; Phillips et al. 2016; Wu et al. 2017). Furthermore, metabolic abnormalities have also been reported in the occipital regions in HD (Martin et al. 2007; Feigin et al. 2001; Reetz et al. 2012). Histologically, a study in HD found that atrophy of the occipital lobe was most pronounced compared to other cortical areas (Lange 1981) and a more recent post-mortem study confirmed reductions in the absolute nerve cell number of the occipital lobe in HD (Rub et al. 2015). The *in vivo* microstructural properties of the occipital cortex have, however, not been a primary focus in HD research to date (Tabrizi et al. 2009, 2012; Henley et al. 2009; Rosas et al. 2008; Hobbs et al. 2010; Muhlau et al. 2007; Martin et al. 2007; Feigin et al. 2001; Reetz et al. 2012; Lange 1981; Coppen et al. 2016; Dogan et al. 2015; Ciarochi et al. 2016; Johnson et al. 2015; Rub et al. 2015; Sotak 2004; Bourbon-Teles et al. 2017; Harrington et al. 2016; Matsui et al. 2015; Phillips et al. 2016; Shaffer et al. 2017; Wu et al. 2017).

As carriers of a CAG repeat ≥ 40 within the mutant gene are certain to develop Huntington’s disease provided they live long enough, carriers in the phase before disease presentation could be examined to explore inevitable changes occurring while progressing towards disease manifestation. Viable markers representing disease progression in HD and its premanifest stage (preHD) are still needed in order to investigate potential intervention effects. To this end, various imaging techniques are being used in biomarker research settings. One such technique is diffusion MRI, where measures can be obtained based on the diffusion characteristics of water molecules in tissues. This, in turn, provides indirect information regarding the microstructure of these tissues (Basser et al. 1994; Jones and Leemans 2011). Potential associations between disease state on the one hand and divergent longitudinal differences in diffusivities on the other hand, could give a tool for quantifying disease progression.

We previously explored whole-brain and striatal diffusivities in (pre) HD and healthy controls, where we found no evidence for significant longitudinal differences between the groups (Odish et al. 2015). Other research groups have more recently demonstrated significant longitudinal differences in various white matter tracts between the groups (Harrington et al. 2016; Shaffer et al. 2017), where interestingly Harrington et al. (2016) found differences only in the superior fronto-occipital fasciculus. Furthermore, recent cross-sectional studies have shown abnormalities related to the occipital regions, such as in white matter projections to the occipital lobe (Matsui et al. 2015), in superficial white matter (Phillips et al. 2016) and in deep white matter tracts of the occipital lobe (Wu et al. 2017).

Given the mounting evidence pointing to an early and preferential involvement of the occipital regions in HD (Tabrizi et al. 2009, 2012; Henley et al. 2009; Rosas et al. 2008; Hobbs et al. 2010; Muhlau et al. 2007; Martin et al. 2007; Feigin et al. 2001; Reetz et al. 2012; Lange 1981; Coppen et al. 2016; Dogan et al. 2015; Ciarochi et al. 2016; Johnson et al. 2015; Rub et al. 2015; Harrington et al. 2016; Matsui et al. 2015; Phillips et al. 2016; Wu et al. 2017), this study aimed to investigate diffusion measures of the occipital cortex in premanifest and early manifest HD and matched healthy controls and explore potential differences in longitudinal changes between the groups and associations of changes herein with clinical and behavioral measures.

## Materials and methods

Procedures regarding participant recruitment, inclusion criteria and clinical measures administered have been previously described in detail (Tabrizi et al. 2009; Odish et al. 2015). In summary, 56 subjects at the Leiden site of the prospective international TRACK-HD study completed a brain MRI scan at baseline and a second scan two years later. The between-scan interval in months is shown in Table 1, without significant between-group differences. The group consisted of 24 healthy controls (49.0 ± 8.2 years), 22 preHD (43.6 ± 8.7 years) and ten early manifest HD (50.2 ± 9.3 years) (Table 1). As previously applied by Tabrizi et al. (2009), to assess the effect of expected proximity to disease onset on diffusion parameters, the preHD group was divided at baseline according to the median (10.9 years) for the predicted years to disease onset into preHD-A (≥ 10.9 years. Mean ± SD: 14.9 ± 4.7) and preHD-B (< 10.9 years. Mean ± SD: 8.6 ± 1.8). The predicted years to disease onset were calculated using the Langbehn method (Langbehn et al. 2004). This resulted in two groups each consisting of eleven subjects (Table 1). The Symbol Digit Modalities Test (SDMT) and the Stroop Word Reading (SWR) task, where visual processing is required, were administered to evaluate potential associations between these commonly used and sensitive longitudinal neurocognitive measures in HD (Tabrizi et al. 2012) and occipital diffusivities. To monitor disease state, the following clinical measures were further evaluated longitudinally for all groups: Unified Huntington’s Disease Rating Scale (UHDRS-TMS), Total Functional Capacity (TFC) and Beck Depression Inventory-II (BDI-II) scores. The study was approved by the Medical Ethics Committee of the Leiden University Medical Center and written informed consent was obtained from all participants.

**Table 1:** Group demographics with clinical and behavioral scores

		Healthy controls	preHD (A and B)	preHD-A	preHD-B	Manifest HD
N		24	22‡	11	11	10
Gender male/female		11/13	9/13	4/7	5/6	4/6
Age in years (at V1), mean (SD)		49.0 (8.2)	43.6 (8.7)	44.2 (5.7)	43.0 (11.2)	50.2 (9.3)
Handedness R/L		20/4	18/4	9/2	9/2	9/1
Level of education (ISCED), median (range)		4 (3)	4 (3)	4 (3)	4 (3)	4 (3)
DART-IQ, mean (SD)		105.0 (9.4)	100.5 (11.2)	101.3 (9.7)	99.6 (13.0)	101.8 (13.5)
CAG repeat length, mean (SD)		n/a	42.6 (2.7)	41.3 (1.4)	43.9 (3.1)^	42.5 (1.2)
Estimated years to onset, mean (SD)		n/a	11.8 (4.7)	14.9 (4.7)	8.6 (1.8)^	n/a
Total functional capacity, mean (SD)						
	V1	13.0 (0.2)	12.8 (0.5)	12.7 (0.7)	12.8 (0.4)	11.0 (1.5)Φ
	V2	12.9 (0.5)	12.6 (0.9)	12.7 (0.6)	12.5 (1.0)	10.3 (2.2)Φ
UHDRS-TMS, mean (SD)						
	V1	2.6 (2.5)	2.6 (1.5)	2.0 (1.5)	3.1 (1.2)	14.6 (7.7)Φ
	V2	2.1 (1.6)	5.7 (5.1) ¥	3.5 (2.2)	8.3 (6.1)*^	23.0 (12.1)Φ
SDMT, mean (SD)						
	V1	49.4 (8.9)	50.1 (11.0)	53.5 (9.3)	46.7 (11.9)	41.2 (9.2)Φ
	V2	50.9 (9.3)	50.6 (10.0)	54.7 (10.0)	46.6 (8.5)^	39.2 (10.6)Φ
SWR, mean (SD)						
	V1	100.1 (13.2)	91.9 (14.2)*	95.6 (9.6)	88.3 (17.3)*	87.7 (14.7)*
	V2	102.0 (15.6)	87.9 (15.7)*	91.4 (9.4)	84.4 (20.0)*	86.4 (18.6)*
BDI-II, mean (SD)						
	V1	4.1 (4.4)	6.4 (6.4)	4.9 (6.0)	7.9 (6.8)	10.2 (8.2)*
	V2	3.9 (4.1)	5.1 (5.6)	3.2 (4.9)	6.9 (5.9)	8.2 (8.4)
Between-scan interval in months, mean (SD)		23.0 (0.8)	23.0 (0.7)	23.2 (0.6)	22.7 (0.7)	23.5 (0.7)

*N* number of participants, *SD* Standard deviation, *n*/*a* not applicable, *ISCED* International Standard Classification of Education, *DART-IQ* Dutch Adult Reading Test Intelligence Quotient, *CAG* Cytosine-Adenine-Guanine, *UHDRS-TMS* Unified Huntington’s Disease Rating Scale-Total Motor Score, *SDMT* Symbol Digit Modalities Test, *SWR* Stroop Word Reading task, *BDI-II* Beck Depression Inventory-II, *V1* visit 1, *V2* visit 2

Significance at p ≤ 0.05 level: * significantly different from controls, Φ significantly different from controls and preHD, ¥ significantly different from controls and HD, ^ significantly different from preHD-A

‡ Including five subjects progressing to the early manifest stage during the two year follow-up period

### Magnetic resonance imaging acquisition

MRI acquisition was performed with a 3-Tesla whole-body scanner (Philips Achieva, Healthcare, Best, The Netherlands) with an eight channel SENSE head coil. T1-weighted image volumes were acquired using a 3D MPRAGE acquisition sequence with the following imaging parameters: TR = 7.7 ms, TE = 3.5 ms, FOV = 24 × 24 cm^2^, matrix size 224 × 224, number of slices = 164, slice thickness = 1.00 mm, and no slice gap. A single-shot echo-planar diffusion tensor imaging sequence was applied with 32 measurement directions and the following scan parameters (Jones and Leemans 2011): TR = 10,004 ms, TE = 56 ms, FOV = 220 × 220 mm^2^ with an acquisition matrix of 112 × 110, 2.00 mm slice thickness, transversal slice orientation, no slice gap, flip angle = 90°, reconstruction voxel dimensions of 1.96 × 1.96 × 2.00 mm^3^, number of slices = 64, b-value = 1,000 s/mm^2^, halfscan factor = 0.61. Parallel imaging (SENSE) was used with a reduction factor of two, NSA = 1, and fat suppression was applied. DTI acquisition time was 6.55 min.

### Image processing

DTI data were analysed using the diffusion MR toolbox ‘ExploreDTI’ Leemans et al. (2009), as previously described (Odish et al. 2015). Automated atlas-based analysis (Kersbergen et al. 2014) using the LPBA40 parcellation map from the SRI24 atlas (Rohlfing et al. 2010) (available at http://www.nitrc.org/projects/sri24/) was performed using affine and elastic registration based on ‘Elastix’ (Klein et al. 2010). All DTI data were visually checked in terms of quality of tensor estimation and quality of registration. As no significant differences were found between hemispheres, left and right hemisphere values of mean diffusivity (MD), axial diffusivity (AD) and radial diffusivity (RD) were calculated and averaged per occipital region as provided by SRI24/LPBA40 (Rohlfing et al. 2010). To correct for multiple comparisons (three occipital regions), a Bonferroni corrected p-value ≤ 0.017 (0.05/3) was considered significant for omnibus F-tests. As fractional anisotropy is not an informative measure in cortical grey matter regions (Beaulieu 2002; Jones et al. 2013), MD, AD and RD are reported.

### Statistical analysis

We used linear mixed models (in R version 3.0.0, R Foundation for Statistical Computing, Vienna, Austria) to model the outcome variables with patient as a random factor to accommodate the within-person repeated nature of the data and to assess the effect of group, corrected for age at time of scanning. Correlations between neurocognitive measures and diffusion metrics were tested in the model. Statistical analyses of group demographics were performed with SPSS (version 20, IBM, USA). Distributions and assumptions were checked. Either Analysis of Variance (ANOVA) or Chi-squared tests were applied where this was appropriate. Potential longitudinal change in clinical measures between the groups was also investigated. Difference values were computed and an ANOVA was performed on these delta-scores to evaluate potential group differences. In case of a significant omnibus F-test, exploratory post-hoc analysis using Fisher’s least significant difference was performed to assess which means were significantly different from each other. As absolute values of diffusivities do not convey meaningful information per se, we report percentage change as an informative longitudinal parameter. Supplementary Fig. 1 shows the evolution of the absolute diffusivity levels between the groups and on individual study participant level from the first to the second visit. A statistical power analysis was performed for sample size estimation based on data from our study, with a Bonferroni corrected α = 0.01 and power = 90%. Differences in group demographics between preHD-A and preHD-B were compared using either independent-samples t-tests or Chi-squared tests, where appropriate.

## Results

There were no statistically significant differences in demographic characteristics between the groups. Only a trend towards a difference in age (p = 0.06) was observed. Hence, age was included as a covariate in subsequent analyses. See Table 1 for group demographics and clinical and behavioral scores. The *early HD* group differed significantly at baseline in their performance in SDMT and SWR when compared to both *controls* and *preHD* subjects. For the *preHD* group, a significantly lower baseline score compared to controls was found for SWR. Furthermore, at the second visit, the *preHD-B* group showed a significantly lower SDMT score compared to *preHD-A*. All results presented hereafter are based on the dynamics during the two year duration of the study.

### Superior occipital gyrus diffusivities

**Fig. 1:**
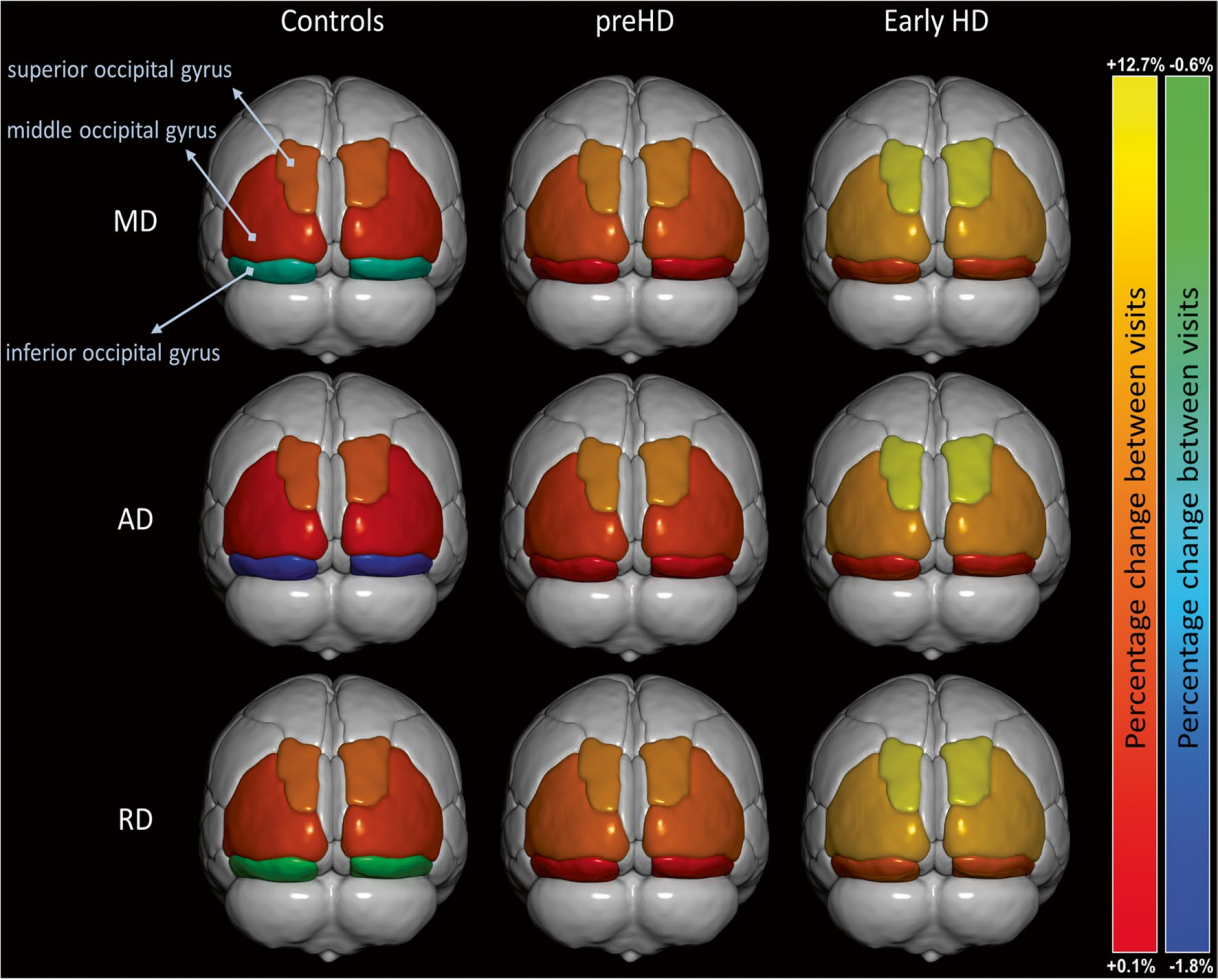
Title: *Longitudinal change in occipital diffusivity values*. Two-year percentage change in mean diffusivity (MD), axial diffusivity (AD) and radial diffusivity (RD) of the three occipital regions of the groups. Significance levels are indicated in Table 2

Longitudinal changes in MD were significantly larger in *early HD* compared to both *preHD* and *controls* (+ 12.3%, + 7.9% and + 6.1%, respectively; p = 0.001). Similar patterns were found for AD (+ 12.7%, + 8.0% and + 5.6%, respectively; p < 0.001) and RD (+ 12.0%, + 7.8% and 6.4%, respectively; p = 0.005) for the three groups. No further longitudinal diffusivity differences in this structure were found upon stratifying the *preHD* group based on expected time to disease onset into *preHD-A* and *preHD-B*. See Table 2 and Fig. 1 for a summary of the results.

**Table 2:** Longitudinal percentage change in diffusion parameters from v1 to v2†

	Controls			preHD (A and B)‡			preHD-A			preHD-B			Early HD		
N	24			22			11			11			10		
	SOG	MOG	IOG	SOG	MOG	IOG	SOG	MOG	IOG	SOG	MOG	IOG	SOG	MOG	IOG
MD	+ 6.1	+ 3.8	-1.1	+ 7.9	+ 5.4	+ 1.0	+ 8.4	+ 4.4	+ 0.5	+ 7.4	+ 6.2 ^	+ 1.4	+ 12.3 Φ	+ 9.0 Φ	+ 4.6 Φ
AD	+ 5.6	+ 2.7	-1.8	+ 8.0	+ 4.5	+ 0.3	+ 8.8	+ 3.7	+ 0.1	+ 7.2	+ 5.1 ^	+ 0.5	+ 12.7 Φ	+ 8.3 Φ	+ 3.4 Φ
RD	+ 6.4	+ 4.5	-0.6	+ 7.8	+ 5.9	+ 1.4	+ 8.2	+ 4.8	+ 0.8	+ 7.4	+ 6.8 ^	+ 1.9	+ 12.0 Φ	+ 9.4 Φ	+ 5.3 Φ

*SOG* Superior Occipital Gyrus, *MOG* Middle Occipital Gyrus, *IOG* Inferior Occipital Gyrus, *MD* mean diffusivity, *AD* axial diffusivity, *RD* radial diffusivity

† Calculated from mixed model-based estimates of the group means for diffusion measures, corrected for age

‡ Including five subjects progressing to the early manifest stage during the two year follow-up period

Significance at p ≤ 0.017 for the omnibus F-test following Bonferroni correction: Φ significantly different from controls and preHD, ^ significantly different from preHD-A, early HD and controls. The MOG is underlined as a prime region of interest based on these results

### Middle occipital gyrus diffusivities

Longitudinal changes in MD were significantly larger in *early HD* compared to both *preHD* and *controls* (+ 9.0%, + 5.4% and + 3.8%, respectively; p < 0.0001). Similar patterns were found for AD (+ 8.3%, + 4.5% and + 2.7%, respectively; p < 0.0001) and RD (+ 9.4%, + 5.9% and + 4.5%, respectively; p < 0.001) for the three groups. Upon stratification of the *preHD* group based on expected time to disease onset, significantly larger longitudinal changes in *preHD-B* compared to *preHD-A* were found in MD (+ 6.2% vs. +4.4%, respectively; p = 0.03), AD (+ 5.1% vs. +3.7%, respectively; p = 0.04) and RD (+ 6.8% vs. +4.8%, respectively; p = 0.02). See Table 2 and Fig. 1 for a summary of the results (data for preHD-B vs. preHD-A are not shown in figure).

### Inferior occipital gyrus diffusivities

Longitudinal changes in MD were significantly larger in *early HD* compared to both *preHD* and *controls* (+ 4.6%, + 1.0% and − 1.1%, respectively; p = 0.001). Similar patterns were found for AD (+ 3.4%, + 0.3% and − 1.8%, respectively; p = 0.002) and RD (+ 5.3%, + 1.4% and − 0.6%, respectively; p = 0.001). No further longitudinal diffusivity differences were found upon stratifying the *preHD* group based on expected time to disease onset into *preHD-A* and *preHD-B*. See Table 2 and Fig. 1 for a summary of the results.

### Associations between occipital diffusivities and neurocognitive measures

The associations between occipital diffusivities and neurocognitive measures were not statistically different between the *preHD* and *early HD* groups. No significant associations were found between the diffusivities of any of the three occipital structures and SDMT (all p’s > 0.05). The SWR showed strong associations with the AD of the Superior Occipital Gyrus (SOG) (p = 0.005), and the MD (p = 0.01), AD (p = 0.009) and RD (p = 0.01) of the Inferior Occipital Gyrus (IOG). No significant associations with any of the diffusivities of the MOG and neurocognitive measures were present. See Table 3 for a summary of the significant associations.

**Table 3:** Associations between occipital diffusivities and neurocognitive measures

	Diffusion parameter	SWR score	*P*
AD-SOG	↓1.8%	↑10 points	0.005
MD-IOG	↓1.2%	↑10 points	0.011
AD-IOG	↓1.1%	↑10 points	0.009
RD-IOG	↓1.3%	↑10 points	0.013

*SOG* Superior Occipital Gyrus, *IOG* Inferior Occipital Gyrus, *MD* mean diffusivity, *AD* axial diffusivity, *RD* radial diffusivity

This table is valid for all participants with a CAG repeat expansion included in the study, as no specific group effects were found on correlations between diffusion parameters and neurocognitive measures. Only significant correlations are shown. ↓ = decrease, ↑ = increase

### Power analysis

Power analysis using these results show that a minimum of 9 subjects per group would be needed to detect a significant longitudinal difference in diffusivity values in 2 years within the occipital cortex (90% power and α = 0.01). There were no significant differences in power between the different diffusivity measures. However, the MOG was the region most prone to longitudinal alteration, thereby most sensitive to demonstrating change. The minimum number of subjects needed to find statistically significant longitudinal difference in the diffusivity of the three occipital regions was as follows: SOG 14, MOG 9 and IOG 12.

## Discussion

We investigated longitudinal microstructural property changes of the occipital cortex in HD. Using a fully automated procedure, we revealed highly divergent longitudinal quantitative imaging measures between preHD, early HD and controls. Associations were found between diffusivity change rates and disease stage in the preHD and early HD groups, providing evidence for an accelerated rate of change correlated with disease progression. Significant correlations between behavioral measures and diffusivity changes in HD were found.

Differences observed in the rate and significance of longitudinal change of SOG, MOG and IOG diffusivities were similar for all measures tested (MD, AD and RD). As such, it does not seem of added value to assess these different diffusivity values individually. However, some of the associations found with cognitive functions were present only with specific measures, for example the inverse relationship found between the Stroop Word Reading task and the AD of SOG. Therefore, it would seem useful to further examine the behavior of the separate diffusion measures in future investigations, as this may provide specific associations with cognitive tests. In preHD, only changes in diffusivities of the MOG could significantly differentiate between preHD-B compared to preHD-A and the other groups. This structure might thus be preferentially affected in the premanifest phase of HD and, in light of these results, could be viewed as a prime region of interest for neuroimaging change within the occipital cortex in preHD. Our power analysis also demonstrated that the MOG is the most sensitive structure of the three examined in detecting longitudinal change between the groups.

The occipital cortex is deservingly gaining interest in HD research. Previous, often serendipitously found alterations in this region (Dogan et al. 2013) increasingly pointed to this structure as relevant in the neuropathology of HD. This study provides strong evidence for a highly differential longitudinal change of diffusion measures in this structure between the studied groups. The relatively short time-frame of the study concomitant with a relatively high rate of change, makes it likely that these disease-related changes could also be reproduced in shorter study intervals, making these measures potentially suitable to use as outcome parameters in shorter clinical trials. These results also pave the way for further investigations into the underlying mechanisms with which the occipital cortex is affected in HD and what the clinical relevance is. Although no specific visual symptoms are known to exist in HD, performance on cognitive tasks examining visuospatial and visuomotor function is known to be reduced in the disorder (Tabrizi et al. 2013; Say et al. 2011). A study investigating the cross-sectional relationship between visual area resting state functional MRI (RS-fMRI), volumetric changes, and cognitive function revealed differences between HD and controls with significant cognitive correlations to visual area RS-fMRI (Wolf et al. 2014). It is further known that impaired emotion recognition is a feature of preHD and early HD (see (Henley et al. 2012) for a systematic review), and results from a previous task-based functional MRI study in preHD revealed reduced neuronal activity in various regions during emotion processing, including the MOG studied in the present report (Novak et al. 2012).

Previous longitudinal reports using diffusion MRI in HD provide heterogeneous findings (Odish et al. 2015; Vandenberghe et al. 2009; Weaver et al. 2009; Sritharan et al. 2010; Poudel et al. 2014; Harrington et al. 2016; Shaffer et al. 2017). Using a tract-based spatial statistics (TBSS) approach, Weaver et al. (2009) compared scans from seven controls, four preHD and three manifest HD subjects obtained one year apart. Significant longitudinal decreases in white matter fractional anisotropy (FA) and AD in the seven mixed preHD and manifest HD group were found compared to the healthy controls. In the study by Sritharan et al. (2010), a region of interest approach was used to investigate several regions of the brain in 17 controls and 18 manifest HD subjects over a one year period, where no significant longitudinal differences in MD were found. Vandenberghe et al. (2009) also applied a region of interest approach in eight manifest HD subjects over a two year period, where no longitudinal differences between the groups were found in MD. In our previous histogram-based study, both global and striatal differences in cross-sectional diffusivities between preHD, early HD and controls were observed, without evidence for any longitudinal differences (Odish et al. 2015).

Using TBSS, a study by Poudel et al. (2014) provided evidence for a significantly increased rate of longitudinal change in FA of the corpus callosum and cingulum of HD patients compared to preHD and controls. Also applying TBSS, Harrington et al. (2016) demonstrated significant longitudinal differences in MD of the superior fronto-occipital fasciculus between preHD and controls using a cohort from the prospective international Predict-HD study (Paulsen et al. 2008). It should be noted, however, that the definition of the premanifest phase in the aforementioned study is different than in our study, making a direct comparison difficult. In the study of Harrington et al. mutant gene-carriers scoring more than 5 points on the UHDRS-TMS were also included to the preHD group, as long as a diagnostic confidence level of 4 was not reached, a level in which an examiner had to have ≥ 99% confidence of seeing unequivocal signs of HD. In our clinical phenotypic characterization of preHD, mutant gene carriers had an UHDRS-TMS of ≤ 5, making the selection much more stringent and the results of the “preHD” group not comparable. Another study by Shaffer et al. (2017) demonstrated longitudinal differences in cortico-striate tracts using a whole brain tractography approach in a larger cohort of preHD subjects from the same Predict-HD study. The inconsistencies in the literature might very well be attributed to inconsistencies in defining the regions/tracts of interest, not selecting the regions/tracts of interest most prone to change, variations in the definition of the premanifest phase, and/or other methodological limitations, such as for TBSS (Bach et al. 2014).

This present study investigates cortical grey matter, where FA is generally not informative (Beaulieu 2002; Jones et al. 2013) and where MD, AD and RD were derived instead. Although the underlying structures studied by Poudel et al. (2014) are different than in this study, one of the goals in HD biomarker research remains to identify the most sensitive longitudinal tools differentiating between preHD, early HD and healthy controls. The annualized rates of diffusivity measure changes in white matter microstructure found by Poudel et al. (2014) were between 1.5%-3.5%, which given the period in the present study would roughly translate into a 3%-7% change rate. Also, no evidence was found for a longitudinal difference in diffusivity change for the preHD group in that report. The rates of change found in the present study are generally more prominent compared to those reported by Poudel et al. (2014). Moreover, a distinct longitudinal diffusivity change was demonstrated in preHD-B, implying that investigating the occipital cortex as a region of interest may provide a more sensitive way to track disease advancement in preHD compared to the corpus callosum and/or cingulum. An important quality for a robust biomarker is reproducibility of results. This makes unbiased, fully automated approaches desirable in order to investigate the effect of an intervention within and between centers as easily and reliably as possible.

Inference of biological meaning based on the observed changes in diffusivity is challenging, especially in grey matter (Beaulieu 2002; Jones et al. 2013). Therefore, caution should be taken when attempting to interpret these results in the light of a disease-specific microstructural effect on the occipital cortex. The findings of small changes in diffusivity values within the healthy control group in the two-year between-scan interval is most likely explained by natural, ongoing, age-related processes of the brain (Hsu et al. 2008, 2010). It is likely that the findings of increased changes in the diffusivities of both preHD and early HD subjects reflect progressive disruption of cell boundaries in this cortical region with disease advancement, causing an increase in tissue permeability and interaxonal spacing due to neural tissue loss (Tellez-Nagel et al. 1974; Sotak 2004). Evidence of ongoing macrostructural neurodegeneration in HD is already known from previous MRI volumetric investigations (Tabrizi et al. 2009, 2012; Henley et al. 2009; Rosas et al. 2008; Hobbs et al. 2010; Muhlau et al. 2007; Vandenberghe et al. 2009; Coppen et al. 2016; Ciarochi et al. 2016; Johnson et al. 2015). The value of the current results lie in the high rate of observed microstructural changes that is disease stage-specific. Potential effects of a therapeutic agent could theoretically be examined by concomitant monitoring of the rate of change in microstructural integrity of the occipital cortex, thereby inferring potential protective effects.

Strengths of this study include a longitudinal design specifically focused on DTI measures obtained from the occipital cortex in HD. Also, an automated atlas-based procedure was applied, which has already shown to provide objective and reproducible results in the clinical setting (Kersbergen et al. 2014). Furthermore, between-scan intervals were alike between all the groups and the same scanner and scan protocol were used at both time points, reducing test–retest variation in DTI data (Takao et al. 2012). Potential limitations of this study include the relatively small sample size of early HD patients and potential imperfect atlas-based segmentations of the occipital cortex (Kersbergen et al. 2014). Notwithstanding these concerns, these results provide evidence for a robust effect on longitudinal diffusivity measures in HD.

## Conclusions

Findings in this study reinforce previous research of disease-stage related occipital involvement in HD, adding evidence for a divergent longitudinal evolution of diffusion measures reflecting microstructural change compared to healthy controls. The results were complemented by significant associations between diffusion measures and SWR, a cognitive task frequently administered in HD research. Investigating the occipital cortex with DTI measures seems to be a promising and sensitive tool to assess the efficacy of future planned disease modifying clinical trials in premanifest and early manifest HD.

## Electronic supplementary material

Below is the link to the electronic supplementary material.

Title: *Longitudinal change in absolute occipital diffusivity values*. Two-year absolute change in mean diffusivity (MD), axial diffusivity (AD) and radial diffusivity (RD) of the three occipital regions of the groups. MD, AD and RD in mm^2^/s (shown x10^3^ for readability). Standard error bars are also shown. V1 = visit 1, v2 = visit 2 (TIF 315 KB)

## Glossary

AD: axial diffusivity
DTI: diffusion tensor imaging
HD: Huntington’s disease
IOG: inferior occipital gyrus
MD: mean diffusivity
MOG: middle occipital gyrus
preHD: premanifest Huntington’s disease
RD: radial diffusivity
SDMT: Symbol Digit Modalities Test
SOG: superior occipital gyrus
SWR: Stroop Word Reading
