# Supplementary figures and images for: Progressive microstructural changes of the occipital cortex in Huntington’s disease
Source: Brain Imaging Behav. 2018 Feb 28;12(6):1786–94. doi: 10.1007/s11682-018-9849-5 (PMC6302057; doi:10.1007/s11682-018-9849-5)

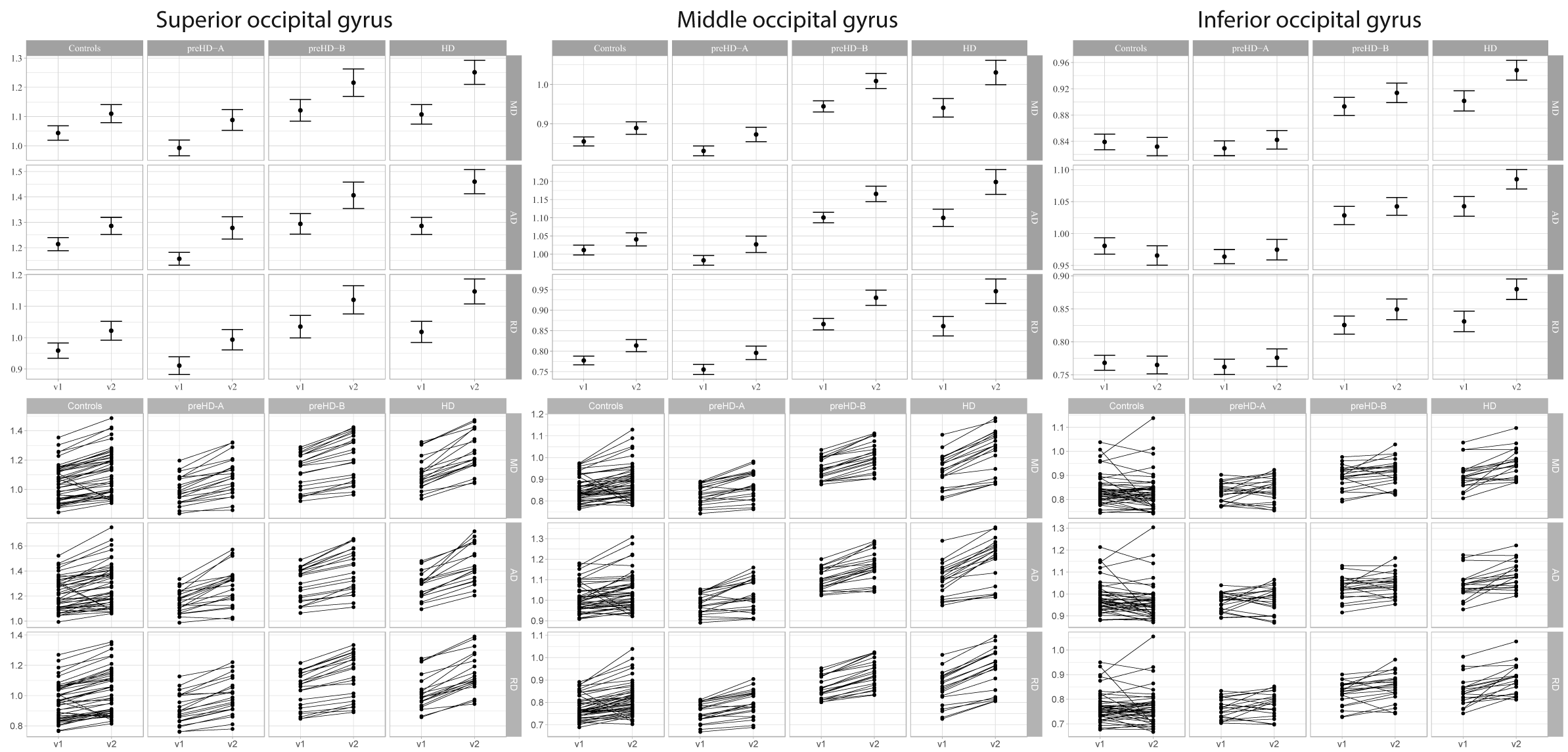

Supplement: Supplementary file 1 — Title: Longitudinal change in absolute occipital diffusivity values. Two-year absolute change in mean diffusivity (MD), axial diffusivity (AD) and radial diffusivity (RD) of the three occipital regions of the groups. MD, AD and RD in mm2/s (shown x103 for readability). Standard error bars are also shown. V1 = visit 1, v2 = visit 2 (TIF 315 KB) [file 11682_2018_9849_MOESM1_ESM.tif]
